# Is There a Role for Immunostimulant Bacterial Lysates in the Management of Respiratory Tract Infection?

**DOI:** 10.3390/biom14101249

**Published:** 2024-10-02

**Authors:** Mario Di Gioacchino, Francesca Santilli, Andrea Pession

**Affiliations:** 1 Institute for Clinical Immunotherapy and Advanced Biological Treatments, 65100 Pescara, Italy; 2 Center for Advanced Science and Technology (CAST), G. d’Annunzio University, 66100 Chieti, Italy; francesca.santilli@unich.it; 3 Department of Medicine and Science of Aging, G. d’Annunzio University, 66100 Chieti, Italy; 4 Department of Medicine and Surgery, “Alma Mater Studiorum”-University of Bologna, 40100 Bologna, Italy; andrea.pession@unibo.it

**Keywords:** Bacterial Lysates, innate immune system, adaptive immune system, respiratory tract infection, asthma, COPD, children, elderly

## Abstract

Bacterial Lysates are immunostimulants clinically prescribed for the prevention of respiratory tract infections (RTIs). It has been shown that Bacterial Lysates upregulate the immune system, acting both on innate and adaptive reactions. In fact, there are demonstrations of their efficacy in restoring the integrity and immune function of epithelial barriers, activating ILC3 and dendritic cells with an enhanced Th1 response, and producing serum IgG and serum and salivary IgA specific to the administered bacterial antigens. The activated immune system also protects against other bacteria and viruses due to a trained immunity effect. Most studies show that the number of RTIs and their severity decrease in Bacterial Lysates-pretreated patients, without relevant side effects. The Bacterial Lysates treatment, in addition to reducing the number of RTIs, also prevents the deterioration of the underlying disease (i.e., COPD) induced by repeated infections. Despite these positive data, the most recent meta-analyses evidence the weakness of the studies performed, which are of low quality and have an inadequate number of patients, some of which were non-randomized while others were without a control group or were performed contemporarily in different clinical conditions or with different ages. The high heterogeneity of the studies does not allow us to state Bacterial Lysates’ effectiveness in preventing RTIs with sufficient certainty. To completely define their indications, double-blind, placebo-controlled, multicenter, randomized clinical trials should be performed for each product and for each indication. The study population should be adequate for each indication. For this purpose, an adequate run-in phase will be necessary.

## 1. Introduction

Respiratory tract infection (RTI) is defined as any infectious disease of the upper or lower respiratory tract. Upper respiratory tract infections (URTIs) include the common cold, laryngitis, pharyngitis/tonsillitis, acute rhinitis, acute rhinosinusitis, and acute otitis media. Lower respiratory tract infections (LRTIs) include acute bronchitis, bronchiolitis, pneumonia, and tracheitis. RTIs are the most prevalent infectious diseases and cause millions of deaths annually worldwide [1]. Further, respiratory infections are the main comorbidity factor in chronic obstructive pulmonary disease (COPD), chronic rhinosinusitis, and asthma. In total, 17 billion URTI incident cases [2] and 489 LRTIs [3] were estimated in 2019 globally, with a relevant social impact. In fact, RTIs provoke a high disability-adjusted life-years rate, with a descending trend with increasing age in children and an increasing trend with age in adults [2,3]. The Global Burden of Disease Study in 2016 reported 2,377,697 deaths in people of all ages worldwide, of which 652,572 occurred in children (<5 years of age) and 1,080,958 occurred in adults older than 70 years [4].

The highest incidence of infection observed in early life, in particular in preterm infants, is due to immature immune responses during the transitional postnatal period [5,6,7,8]. The age-related immune dysfunctions associated with inflammaging and the presence of comorbidities contribute to the increased risk of morbidity and mortality from RTIs among the elderly [9,10,11,12]. Moreover, the elderly have a weak response to vaccines and their innate immune system is less able to clear infections [13,14]. The presence of RTI has been reported to increase mortality in old people by 6–7% [15]. Independently of age, chronic airway diseases such as asthma, COPD, or cystic fibrosis are associated with recurrent RTIs (rRTIs), with worsening of their conditions [16,17,18,19,20]. Active and passive smoking [21,22], low serum vitamin D levels [22,23], a lack of physical activity [24], sudden changes in temperature at work [25], occupational exposure to various physical, chemical, and other hazards [26,27], obesity, and Type 2 diabetes [22,28,29] are reported as risk factors for RTIs. Moreover, immunocompromised individuals (including those with immunodeficiency, cystic fibrosis, and HIV, as well as the use of corticosteroids, transplantation, and post-splenectomy) [30,31,32,33,34,35,36,37,38,39,40] and those with anatomical anomalies including facial dysmorphic changes [41] or nasal polyposis [42] are also at high risk for RTI.

The use of antibiotics remains the most efficacious treatment of bacterial RTIs and accounts for 60% of all antibiotics prescribed [43,44,45]. Antibiotic use, in addition to hygiene measures, could reduce under-5 deaths in countries with the highest LRTI burden [3]. However, despite antibiotics, bacterial infections are still a severe health problem because of the emergence of resistant bacteria, antibiotic overuse and misuse, and the lack of new drug development [46,47]. Antibiotic resistance leads to higher medical costs, prolonged hospital stays, and increased mortality. On the other hand, there is evidence that antibiotics have limited efficacy in treating a large proportion of RTIs [48], including acute otitis media, acute cough/acute bronchitis, acute sore throat/acute pharyngitis/acute tonsillitis, acute rhinosinusitis, and the common cold. Moreover, in an international prospective study, van Houten et al. [49] evidenced major antibiotic overuse in patients with RTIs due to viral infections in adults. The authors underlined the need for effective interventions to decrease antibiotic overuse in RTI patients of all ages. The inappropriate prescribing of antibiotics has the potential to cause drug-related adverse events (diarrhea, nausea and vomiting, dizziness, yeast infections, hypersensitivity, and adverse reactions to antibiotics).

Prevention should be the key intervention to address RTI incidence and the frequent consequent disabilities and mortality, with vaccines being the most promising strategy [3,50]. However, there is still a lack of vaccines against most of the infectious agents responsible for RTIs, so other prophylactic strategies must be developed [51,52]. Bacterial Lysates were introduced in the 1970s as oral vaccines for the prevention and treatment of RTIs [52,53,54] and are currently used in many countries worldwide.

In September 2019, the European Medicines Agency stated that there is “… some evidence of effectiveness of these medicines in the prevention of recurrent respiratory tract infections and the safety profile is in line with what is expected for this type of product”. The Committee for Medicinal Products for Human Use therefore recommended that use of BLs for prevention can continue, but the companies must provide further data on safety and effectiveness from new clinical studies by 2026” (clinical studies were postponed by 2 years due to the COVID pandemic) [55].

In this review, the authors summarize the existing literature concerning the mechanisms of actions and the clinical efficacy of Bacterial Lysates, reporting both the substantial homogeneity of the positive results and the weakness of the studies performed.

## 2. Bacterial Lysates—Definition, Main Bacterial Species, and Processing Methods

Bacterial Lysates are mixtures of antigens derived from inactivated pathogens frequently involved in RTIs. The European Medicines Agency defines Bacterial Lysates as medicines made from bacterial cells that are broken down and are intended to stimulate the immune system to recognize and fight infections.

Bacterial lysis is obtained by chemical (alkaline) or mechanical treatment, while heat and detergents have also been used. The antigenic structure seems to be better maintained by mechanical disruption, while chemical lysis produces denaturation of bacterial antigens. Obtained antigens are then mixed in specific proportions to obtain a polyvalent Bacterial Lysate [56]. Commercially available Bacterial Lysates are listed in Table 1. The various procedures for lysis, not completely available to the research community, should be appropriately reported and studied to allow the scientific community to verify the possible antigenic alteration. On the other hand, there are no standardized protocols for the various methods of lysis, and this makes their reproducibility challenging, with difficulty in evaluating the class effect of the various preparations and comparing the immunological and clinical effects of Bacterial Lysates.

## 3. Mechanisms of Action of Bacterial Lysates

The rationale for using Bacterial Lysates for the prevention of rRTIs lies in the hypothesis that they mimic natural exposure to microbes, promoting an innate, adaptive, antigen-specific immune response against the administered antigens. Bacterial Lysates can be administered orally, intranasally, or sublingually; the latter two directly activate local mucosal immunity at the level of the respiratory epithelium and submucosa [57]. The activity of the first route lies in the known interaction between the intestinal and bronchial immune system (the so-called gut–lung axis) [52,58,59,60,61]. Immune cells activated in the intestinal mucosa migrate through mesenteric lymph nodes, blood, and lymph to the lymphoid tissue of the bronchial mucosa. Bacterial antigens administered through Bacterial Lysates act as pathogen-associated molecular patterns on innate immunity and as antigens on adaptive immunity [60,62]. Bacterial Lysates prime the immune system, activating antigen-presenting cells such as macrophages and dendritic cells (DCs) and epithelial cells through TLR-2 and TLR-4 receptors [62,63,64,65,66,67]. As a consequence, mature-activated DCs produce IL-1, IL-6, and TNF-α (inflammatory cytokines) [63] and secrete IFN-α, IFN-γ, IFN-β [68], IL-8, CCL2, and CCL20 [69] (inflammatory chemokines). This results in increased cytotoxicity, in particular with an increase in CD8 cells, and the activation of NK cells and phagocytic activity [66,67]. Moreover, BL-activated alveolar macrophages produce Il6, Il1b, TNF-α, inducible nitric oxide synthase, and antimicrobial peptides Lcn2, CAMP, and Saa3 [70]. DCs are also essential players in adaptive immunity, acting as antigen-presenting cells. BLs/TLR binding activates DCs, resulting in the up-regulated expression of MHC II, CD40, CD86, and HLA-DR and the de novo expression of CD83. DCs migrate to lymph nodes where they interact with T cells [71,72,73,74,75]. This is followed by the activation of T cells and differentiation of B cells into plasma cells, with the increased production of antigen-specific immunoglobulins [74,76,77]. In particular, it has been shown that intranasal Bacterial Lysate administration enhances IgA concentrations in the respiratory mucosa, with consequent protection against respiratory microbes [78,79,80]. The BL-induced immune response has a Th17/Th1 profile, with increased production of IL-17A and IFN-γ and decreased IL-4 levels [63,70].

The activation of innate immune cells allows protection against other bacteria and viruses in addition to those specifically administered with Bacterial Lysates. BL-activated bronchial and alveolar epithelial cells participate in this immune protection [81]. In fact, BL-stimulated alveolar and bronchial epithelial cells highly express several pro-inflammatory chemokines (Cxcl1, Cxcl8, Ccl20, Cxcl11, Ccl2, and Ccl5) and cytokines (IL6 and TNF-α) [70] and the novo expression of beta defensin 1, a major antimicrobial peptide [70,82]. Moreover, BL-stimulated bronchial epithelial cells release IL-23, which induces IL-22 production by innate lymphoid cells, in turn favoring the release of antimicrobial peptides by bronchial epithelial cells [82].

Bacterial Lysate administration also has anti-viral activities, inducing both DCs to produce anti-viral cytokines/chemokines (IFN-β, IFN-γ, IL-8, CCL2, CCL20, and TNF-α) [83] and polyclonal activated B cells to produce IgA and IgG specific to the influenza virus and the respiratory syncytial virus in animals not previously exposed to these viruses [74]. Increases in virus-specific IgA and IgG were also seen in children affected by bronchiolitis after treatment with Bacterial Lysates [84]. Moreover, BL-activated NK cells are involved in the elimination of virus-infected cells [85]. On the other hand, it has been demonstrated that treatment with Bacterial Lysates preventing RTI inhibits the “vicious circle” of bacteria/virus interaction [86].

**Figure 1:**
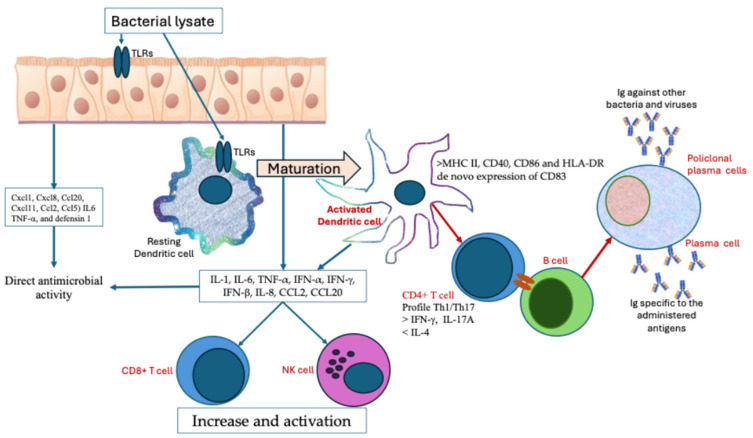
Bacterial Lysates stimulate epithelial cells and dendritic cells through TLR 2 and 4. DCs become activated and secerning cytokines and chemokines stimulate CD8 and NK cells to proliferate and activate. The interaction of DCs with T lymphocytes induces the proliferation of B cells that maturate to plasma cells secerning immunoglobulins specific for the administered Bacterial Lysate. Polyclonal plasma cells produce Ig against other bacteria and viruses. BL-activated epithelial cells secrete chemokines, cytokines, and β-defensin 1 with antimicrobial activity.

Some authors have suggested that taken together, the activation of the innate and adaptive immune system generates a state of “pre-alert” against infection [80]. It is the concept of “trained immunity-based vaccines” that generates a strong immune stimulus, which increases the nonspecific response of innate immune cells and strengthens the activated state of dendritic cells to enhance adaptive T cell responses to specific and bystander antigens [87,88]. Figure 1 summarizes the immune mechanisms of Bacterial Lysates.

The lungs have tertiary lymphoid structures, bronchus-associated lymphoid tissue (BALT), which consists of lymphoid follicles in the lungs and bronchus. BALT is an effective priming site of adaptive B cell and T cell responses directed against airborne antigens. It consists of a stromal cell network, with separated areas for T and B cells, endothelial venules, and lymphatics [89]. Physiologically, BALT is present in the lungs of children to give protection to the diverse respiratory challenges during this formative life stage. In adults, BALT has not been reported in healthy adults but is inducible (iBALT) under pathologic conditions, such as chronic or repeated respiratory infection [90]. IBALT forms in the perivascular space of blood vessels on the basal side of the bronchial epithelium, and consists of a B cell follicle with T cells and DCs surrounding the B cell follicle [91]. There are no specific studies on the direct activity of Bacterial Lysates on BALT or iBALT, as they can be administered only by the oral route (sublingual or gastrointestinal). However, even though the various tertiary lymphoid structures (gastrointestinal, nasal, laryngeal, and pharyngeal) are separated, they communicate through the common mucosal immune system [92]. It follows that lymphocytes induced by a specific antigen at one site can migrate as effector cells, providing protection from the same antigen in other organs. This is understood as the concept of an “integrated immune system” [93].

## 4. In Vitro and Animal Experiments

Research in animals has revealed that Bacterial Lysates act as immunomodulators. Blesser et al. [94] found that Bacterial Lysates in mice increase the production of immunoglobulin specific to the bacterial strains contained in the extract. In the same experiments, all BL-pretreated mice survived after being infected either by Haemophilus influenzae virus or Salmonella typhimurium, with respect to non-pretreated mice. Moreover, in a further experiment, they found that 70% of mice pretreated by Bacterial Lysates and then exposed to a sublethal dose of influenza virus and superinfected by *S. pneumoniae* survived, whereas all non-pretreated mice died after 3 days [95]. Bacterial Lysates have also been evidenced to protect against pneumococcal pneumonia, engaging pro-inflammatory gene expression in macrophages and epithelial cells [70]. Both Bacterial Lysates obtained by chemical or mechanical lysis act as TLR antagonists, inducing NF-kB activation in a MyD88-dependent manner and inducing antibacterial activity in mice lungs [64].

Pretreatment with Bacterial Lysates in primary human bronchial epithelial cells of healthy, asthmatic, and COPD patients infected by rhinovirus reduced the number of infected cells with an increase in their survival with respect to non-treated cultures. Bacterial Lysate activity was supported by the concomitant activation of Erk1/2 MAPK and cAMP signaling in epithelial cells along with an increase in C1q-R and β-defensin [96]. In addition, in murine models, Bacterial Lysates positively modulate the production and release of inflammatory cytokines and the antibody response against both respiratory syncytial virus [97] and influenza virus [74,97] through the activation of DCs via TLR4 [97]. It has been demonstrated that in human immune cells, TLR2 and TLR4 orchestrate the immune response to a Bacterial Lysate, which binds to both receptors [98]. In a further study, a preventive BL airway administration inhibited viral replication in a dose- and time-dependent manner, with an increase in IFN-β gene expression, also preventing the inflammation and limiting the ILC2 recruitment in the airways [99]. Recently, it was evidenced that Bacterial Lysates significantly downregulated ACE2 transcription and expression, inhibiting the SARS-CoV-2 infection of bronchial epithelial cells [100]. Furthermore, there is evidence in animal experiments that Bacterial Lysates induce the prevalence of the Th1/Th17 response with respect to the Th2 type [70] and induce an increase in the expression of T regulatory cells [80,101]. These changes in T cell prevalence suggested the possibility of counteracting the Th2 inflammation typical of an allergic reaction with the administration of Bacterial Lysates. In fact, in animal experiments, Bacterial Lysates prevented allergic sensitization through the activation of IL10-producing T regulatory cells [101,102] and reducing the level of IgE [103]. Moreover, interference with the airway epithelium/IL-33/ILC2 axis and lung allergen-induced TH2 response, along with the induction of tolerogenic dendritic cells, was the underlying mechanism of suppression of alternaria-induced experimental asthma by preventive/concurrent Bacterial Lysate administration during sensitization in mice [104]. Moreover, other authors also evidenced a significant reduction in eosinophilic infiltration, mucus plugs, and airway wall thickness in a model of asthmatic mice after oral administration of Bacterial Lysates with respect to non-treated mice [105].

## 5. Clinical Efficacy of Bacterial Lysates

Clinical trials with Bacterial Lysates, obtained via both chemical and mechanical lysis, were carried out in patients particularly susceptible to RTIs, such as children, the elderly, and COPD patients. A reduction in infection episodes was found in most studies as a clinical consequence of the Bacterial Lysate immunostimulatory effects.

### 5.1. Pediatric Patients

The first trials were conducted in the 1980s and 1990s. All but one showed a reduction in infectious episodes in BL-treated children. In a double-blind clinical trial, the incidence and duration of infectious episodes (and the duration of concomitant treatments) significantly decreased in Bacterial Lysate-pretreated children with rhinosinusitis with respect to controls. The authors evidenced a correlated increase in serum IgA [106]. The risk of suffering from three or more infectious episodes of the upper respiratory tract and one or more episodes of gastroenteritis was reduced by 40% in BL-pretreated patients, as reported in a large trial involving 423 children at high risk of infection, as they attended day-care centers [107]. A further trial evaluated the efficacy of the combination of Bacterial Lysates added to amoxicillin/clavulanate in the treatment of subacute sinusitis with respect to an antibiotic plus placebo. The infectious episodes in the active group lasted less than in the control group treated only by antibiotics, with a subsequent lower incidence of RTIs [108]. The only negative study was performed in 1986 with no significant differences in the number of acute RTIs between children (*n* = 825) receiving intranasal BL treatment and children in the placebo group (*n* = 327) [109].

In the following decades, more trials were performed, all showing the efficacy of Bacterial Lysates in preventing recurrent infections in children. A one-year RCT involving 188 children evidenced a 50% reduction in infections in treated patients with a sustained effect during the 6-month follow-up after the end of the study, without relevant side effects [110]. A further one-year RCT, on children aged 1–12 years with a history of RTIs, evidenced a significant reduction in RTIs and antibiotic use in BL-treated children with respect to the controls [111]. This trial evidenced, as a secondary endpoint, a 68% reduction in acute otitis media in BL-treated children, in agreement with a previous evaluation [112]. Similar results on the prevention of otitis media were obtained by Mora et al. [113]. The preventive effects of Bacterial Lysates in the treatment of children with asthma and RRIs were also observed by Su et al. [114] and Chen et al. [115] in China with randomized placebo-controlled trials.

The data on the effect and safety of Bacterial Lysates in reducing upper RTIs were confirmed by subsequent large RCTs [116,117,118], also showing beneficial effects in children with IgG deficiency [117]. An increase in B-lymphocytes in BL-treated subjects, concomitant with clinical benefit, was seen [118]. A reduction or no episodes of acute tonsillitis were evidenced in a 5-year retrospective cohort study on 177 children with 131 receiving Bacterial Lysates. It is of note that no patients with total success required tonsillectomy in the long-term follow-up [119].

A recent DBPC trial evidenced a significant reduction in day-care absence for children and therefore lost working days for parents, in addition to a significant benefit in the number of RTIs among BL-treated children with respect to those receiving a placebo; the effects were greater in children with a history of frequent RTIs [120]. In a 6-month prospective cohort study involving 57 children (aged 1–9 years), a decrease in the number of RTIs was evidenced, along with a decrease in the severity of symptoms in the absence of adverse effects [121].

A further aspect was evidenced in children, linked to the ability of Bacterial Lysates to influence the Th1/Th2 balance of the immune response in favor of Th1 [70] with a significantly increased release of IFN-γ and IL-12 [63,122,123], enhancing the reaction against microorganisms and weakening the Th2 type response. Moreover, Bacterial Lysates concomitantly induced the differentiation of T regulatory cells [124] that are protective against allergy and asthma. This led some authors to study the effects of Bacterial Lysates on allergy and asthma, with microorganisms, in particular a virus infection, being a trigger for asthma exacerbation and high Th2 and a defective Treg response being characteristic of the allergic sensitization [66,95,125,126]. Moreover, the respiratory syncytial virus and human rhinovirus induce airway inflammation, epithelial damage, and a type 2 immune response. Recurrent infections in children, also facilitated by their physiological immune immaturity, can induce a persistent Th2 prevalent immune reaction, therefore facilitating allergic sensitization [127,128]. The hypothesis was that the ability of BL prophylaxis to prevent recurrent wheezing/asthma attacks lies in the modulation of the immune response against viral infections [59]. The benefit of BL prophylaxis was observed in a randomized, double-blind, placebo-controlled, parallel-group study including 75 children (aged 1–6 years old) with recurrent wheezing and viral infections. The administration of Bacterial Lysates provoked a significant reduction in acute RTIs and a 37.9% reduction in wheezing episodes with respect to the placebo group (*p* < 0.001) [129]. These results were similar to those observed in a previous 12-month study [130] and in a study of school-aged asthmatic children on long-term control with inhaled corticosteroids [131]. These effects were associated with an increase in serum IFN-γ and IL10, demonstrating an immune Th1 deviation and an increase in T regulatory activity [132].

The efficacy of Bacterial Lysates was also evaluated in the primary prevention of lower RT infections in a recent study in at-risk children [133]. The infectious episodes were significantly less frequent and less severe in children treated with Bacterial Lysates with respect to the controls.

Based on the BL interference with the Th1/Th2 balance and their effects on T reg, a large multicenter study was started with the primary objective of evaluating the possible prevention of wheezing and asthma with Bacterial Lysate administration since the first year of life. The study is not yet completed (https://classic.clinicaltrials.gov/ct2/show/NCT02148796, accessed on 3 March 2024).

In fact, recent reviews and meta-analyses [134,135,136,137,138] have reported substantial evidence for the benefits offered by Bacterial Lysates in reducing the risk of RTIs in children, although they all revealed important weaknesses in the conducted studies as reported in the meta-analysis chapter.

### 5.2. Adult Patients

Bacterial and virus infections are increasingly recognized as an independent stimulus to airway inflammation [18], modulating the severity and frequency of COPD and chronic bronchitis exacerbations [139,140,141], which are the main conditions associated with recurrent RTIs. Therefore, the treatment of such infections may also be useful in delaying the evolution of these diseases. One of the first RCTs performed in 1990 [142] involving 265 COPD patients treated by Bacterial Lysates demonstrated a statistically significant reduction in infectious events and a concomitant reduction in the use of antibiotics. Also in the 1990s, double-blind, placebo-controlled trials showed the effectiveness of Bacterial Lysates in reducing the mean score of symptoms, the number, severity, and duration of acute exacerbations, and the risk and number of days of hospitalization for respiratory problems with a reduction in the use of conventional therapy in BL-treated groups with respect to placebo groups [143]. Opposite effects were seen in a very large study conducted in 1997, in which the authors reported no differences in COPD exacerbations, hospitalizations, and the severity of the disease in BL-treated patients with respect to controls [144]. During the 2000s, an RCT confirmed the activity of Bacterial Lysates on BPCO exacerbations and found a significant reduction in antibiotics use in the actively treated group with respect to the placebo group [145]. Solèr et al. [146] confirmed such results in the elderly with chronic bronchitis and COPD, further highlighting that current or past smoking patients saw greater benefits from the treatment. Cazzola et al. [147] studied the effects of the combination of Bacterial Lysates with salmeterol/fluticasone versus an inhalant alone. The trial showed a reduced total number of exacerbations, rate of exacerbations per patient per year, number of exacerbations needing oral corticosteroids, and total number of hospitalizations in BL-treated patients. Genda et al. [148] confirmed the data on exacerbations and hospitalization. In contrast, the DBPC trial [149] conducted with 288 moderate to very severe COPD patients assigned to either Bacterial Lysates or a placebo, in addition to inhalation therapy, did not achieve their primary outcome (a reduction in the number of exacerbations by 25% in the Bacterial Lysate-treated group with respect to the placebo group). However, a reduced number of days with fever, days of hospitalization, and number of days in poor health were observed in the BL-treated group. A meta-analysis published in 2015 concluded that the current evidence did not support a beneficial effect of Bacterial Lysates for COPD patients in terms of the duration of hospitalization and the severity of acute exacerbation [150]. The authors were only able to include five trials in the meta-analysis, with the majority of studies performed until then of very low quality. However, in the following years, a number of trials reported positive results on the efficacy of Bacterial Lysates in COPD patients. A clinical trial on 384 patients showed that 12 weeks of BL treatment significantly reduced the proportion of patients with acute exacerbation with respect to the controls. These benefits were maintained for up to 22 weeks [151]. Positive results were achieved by a further trial on 150 COPD elderly patients regarding the number of acute exacerbations in BL-treated patients with respect to controls. Further, there was evidence that the addition of Bacterial Lysates to the recommended treatment delayed the deterioration in lung function in elderly patients with stable COPD, with a significant difference in lung function parameters between the two groups [152]. These results on clinical and functional parameters were confirmed by an RCT on 60 patients with frequent exacerbations of COPD [153]. Ricci et al. [154] evidenced a reduction in infectious episodes after treatment with Bacterial Lysates through the induction of an antibody-mediated immune response, efficient not only against bacteria present in the product but also against different microbes. A metanalysis published in 2022, with the evidence of these most recent trials, underlined the efficacy of Bacterial Lysates in reducing the exacerbation rate, the mean number of exacerbations, and the severity of the disease in COPD patients, without relevant side effects [155]. However, the heterogeneity in addition to the other important weaknesses of the selected studies meant the data on efficacy were not conclusive (see the meta-analysis chapter).

Susceptibility to RTIs is typical not only of patients with COPD and chronic bronchitis but also of other adult categories, such as the elderly, people living in communities, and patients with immunodeficiency and other chronic pathologies.

In the 1980s and 1990s, a number of clinical studies were performed in non-BPCO adults with RTIs, demonstrating the efficacy of Bacterial Lysates in reducing the number of acute infectious episodes [146,147,148,149,150,151,152,153,154,155,156,157,158] and the number, severity, and duration of upper and lower RTIs [159]. Subjects with purulent sinusitis had lower numbers of infections when pretreated by Bacterial Lysates, with reduced purulent nasal discharge, coughing, and headaches [160]. Positive results on RTIs were also achieved in subjects receiving hemodialysis with a significant protective effect [161]. In the 2000s, an RCT involving 140 adults with RTIs evidenced better effects of Bacterial Lysates compared to placebo on the number and duration of RTIs, working days lost because of infections, and the need for antibiotics. In this study, Bacterial Lysates obtained via mechanical lysis were more efficacious in comparison to those obtained by chemical lysis [162]. A successive study also showed a reduced duration of infectious episodes and a decreased need for antibiotics in patients with autoimmune nephrosis [163]. In an interventional, non-randomized study, treatment with Bacterial Lysates was followed by a reduction in RTI with respect to prior treatment, with reduced need for the use of antibiotics in 104 HIV-infected patients [164]. An interesting study was performed in cloistered nuns with recurrent URTIs. A significant reduction in the number and duration of infections was observed in the active treatment group with respect to the placebo, with an increase in salivary IgA in the active treatment group only [165]. Braido et al. [166] performed a double-blind, placebo-controlled, randomized multicenter trial involving 160 patients (71 in the active group and 81 in the placebo group) with a mean age of 42.4 ± 15.14. Both the primary (the number of RTIs) and secondary endpoints (RTI duration, frequency and severity of the acute episodes, the use of drugs, and the number of missed workdays) were reached, with a high statistical significance compared with the placebo group. The estimated risk of needing antibiotics was reduced by 52.1%. The only limitation of the study was that no discrimination was made between URTIs and LRTIs.

A recent study explored the effect of Bacterial Lysates in the treatment of patients with non-severe community-acquired pneumonia. A group of patients received antibiotic treatment alone, and in the second, third, and fourth groups with Bacterial Lysates, an immunomodulator (azoximer bromide) or placebo was added. The overall duration of all symptoms was lower in the BL/immunomodulator groups compared with the control group, with an improvement in the pro-inflammatory cytokine profile [167]. The clinical benefit lasted the successive 2 years of follow-up.

The work of Pizzimenti et al. [168] is of great interest, which found that a Bacterial Lysate, Lantigen B, modulated the expression of ACE2, hypothesizing the possibility of administering Bacterial Lysates for the prevention of SARS-COV.

## 6. Meta-Analyses on Bacterial Lysates

Here, we report the three most recent meta-analyses published in 2022/2023, the first on the effect of Bacterial Lysates in children [138], the second in adults with COPD [150], and the last specific to one Bacterial Lysate, Lantigen B [169].

The meta-analysis on the effects of Bacterial Lysates in children [138] found only 38 trials with sufficient quality to be evaluated: all but 3 (2017, 2019, and 2020) were performed before 2004, the majority in the 1980s and 1990s. The quantitative analysis indicates that Bacterial Lysates reduce RTI incidence in children by 40% on average, with this observation constant across all studies. However, the authors reported low-quality evidence, substantial heterogeneity, and the possibility of publication bias. The quality of the evidence (GRADE) was low for the number of RTIs and the ratio of mean RTIs, with high heterogeneity in the number of RTIs in the control group, and very low on the adverse effects, which were reported in only 14 trials. Moreover, various studies were performed with subjects with a wide age range (aged 1–18 years) and both URTIs and LRTIs were included. Most studies lasted <6 months. Moreover, the primary endpoints of the trials were diverse. Only 19 trials were conducted with more than 40 patients.

The meta-analysis on the effect of Bacterial Lysates on COPD [155], reporting Bacterial Lysates as useful in reducing exacerbations and symptoms, was based on only twelve studies (nine randomized controlled trials and three abstracts of randomized controlled trials with sufficient data) performed from 1998 to 2020 with sufficient scientific quality, showing a significant clinical efficacy of Bacterial Lysates overall. The duration of the treatment lasted from 12 weeks to 6 months in the various studies, and the follow-up period was 10 weeks to 9 months. Six studies did not report dropouts. The two main biases were the differences in lung function, correlated with exacerbation risk, and the non-standardized symptom evaluation. The severity of COPD varied greatly among the studies with the mean FEV1/predicted ranging from 30% to 85%. Only eight studies reported the rate of exacerbation with detailed numbers and only four reported the hospitalization rate. Moreover, the selected studies did not homogeneously report the efficacy against symptoms (the changes in sputum, cough, severity of dyspnea, and fever). Furthermore, the included studies also had methodological issues such as explicit allocation concealment, which can reduce the strength of the evidence. Finally, some important issues were not evaluated: are Bacterial Lysates effective in preventing exacerbations of COPD in high-risk patients with severely impaired lung function and reducing the need for antibiotics and corticosteroids?

All possible literature on the effects of a single Bacterial Lysate (Lantigen B) was analyzed by Braido et al. in 2023 [169] who obtained the manuscript of the oldest studies, studies published in non-indexed national journals, and unpublished data from the manufacturer. In total, 37 studies were analyzed, of which 22 were eligible for the meta-analysis. A total of 4571 patients (of which 2888 were from a single study conducted in 1981) were evaluated in these studies, with 2421 treated with Lantigen B and 2150 controls. Fourteen studies were performed in adults, seven in children, and one in both. Eight studies were performed in healthy subjects, with the others involving patients with recurrent RTIs. The quantitative analysis showed that all primary outcomes (the number of exacerbations during the study period, days of illness for recurrent RTIs, number of days with fever, and number of days absent from work or school) were met in patients treated with the Bacterial Lysate with respect to the placebo group, with an overall reduction of −47%, evaluating all studies together. The data were similar considering healthy subjects, adults with or without recurrent RTI, and pediatric patients separately. Moreover, the prophylactic effect was lower in healthy subjects with respect to patients with a history of recurrent RTIs.

However, these results should be interpreted with caution. In the qualitative analysis, the authors pointed out the significant heterogeneity of the study methods and the accuracy of the reports. The small sample size of the majority of the studies makes it difficult to analyze the effect of the Bacterial Lysate in different pathological conditions predisposing patients to RTIs. Not all studies shared the same primary outcomes, the follow-up varied from 1 to 7.5 months, and not all studies reported/described the determination of the sample size, randomization procedure, number of dropouts, accurate statistical analysis, and adverse events. These limitations and the high heterogeneity of the studies make it impossible to draw definitive conclusions on the clinical efficacy of Bacterial Lysates in the prevention of RTIs in both adults and children. However, there is strong homogeneity in the observed results despite the large standard errors observed. The authors concluded that, with the frequencies of responses being very constant across the various studies, the clinical efficacy of Lantigen B in RTI prophylaxis in both adults and children with recurrent RTI is highly suggestive.

## 7. Discussion

This analysis of the literature related to the efficacy of Bacterial Lysates allows us to conclude that the data published are in favor of the use of Bacterial Lysates for the prevention of RTIs. The various published reviews focused, in general, on Bacterial Lysates as a class, others on specific Bacterial Lysates, others on RTI prevention in general, and finally, a portion on specific clinical conditions favoring RTIs such as BPCO, immune system immaturity in children, and immune system failure in elderly.

In addition to the partial positive note on clinical efficacy, all authors agreed that there are severe limitations among the trials performed, so there is a need for high-quality, large, multicenter, double-blind, placebo-controlled randomized clinical trials to confirm the role of immunostimulants in preventing RTIs in children as well as in adults, elderly, and COPD patients.

One of the main problems to be addressed in future studies is the determination of the sample size because, after the COVID-19 pandemic period, the number of RTIs has been reduced worldwide with respect to previous periods [170,171,172]. Therefore, there is a need for a correct evaluation of the real incidence of RTIs: the lower the RTI incidence, the higher the sample size of the study.

The trials should be made in selected specific clinical conditions and age groups to allow a well-defined therapeutic indication of the tested product.

Bacterial Lysates can be considered a class of drugs but there are many differences among the various products. Bacterial Lysates have different bacterial strains and are prepared by different methods with consequent diversity in the quality and purity of the product with the presence or absence of other cell constituents such as polysaccharides. Therefore, although different Bacterial Lysates may share some common mechanisms, some results may be specific to certain products.

As we found no study comparing all Bacterial Lysates, it was not possible to establish the superiority of a certain kind of pharmaceutical preparation. It will be useful to compare products with the same bacterial culture made using different procedures. This would allow for choosing the best method for preparing immunostimulant lysates. Moreover, there were no studies comparing the efficacy of the different routes of administration: local (nasal and sublingual) and oral.

Finally, there are insufficient data on the use of Bacterial Lysates in acute infections, immunodeficiencies, autoimmune diseases, and active tuberculosis and cardiopulmonary insufficiency.

In any case, specific vaccines remain the first choice for some bacteria, and an example is that against *Streptococcus pneumoniae*. The are four vaccines for this bacterium, differentiated by the number of serotypes they contain. It has been reported that 60% to 70% of the success in preventing pneumococcal disease is caused by serotypes present in the vaccine [173]. Even this vaccine, as with others, induces a trained immunity against other infections. The effectiveness of a vaccine is therefore higher than that observed with Bacterial Lysates. However, there are some issues to be considered. Vaccination is often followed by an increase in the frequency of infections by serotypes not present in the administered vaccine. Furthermore, it has been reported that there is an increase in antibiotic resistance among nonvaccine serotypes [174].

Furthermore, it will be interesting to study the effects of Bacterial Lysates with molecules stimulating the immune system, such as FLT3L and MALP-2. The first acts as a growth factor on B and T cells by activating the hematopoietic progenitors [175]. It has been shown that Flt3L induced an increase in the number of dendritic cells in nasal-associated lymphoid tissue [176]. The second stimulates the response of macrophages, indirectly activates NK cells, and enhances antigen presentation on dendritic cells [177].

## 8. Conclusions

The present review underlines the major shortcomings of the studies performed on the effects of Bacterial Lysates in the prevention of RTIs. It appears evident that the lack of rigor in experimental design and standardized protocols, insufficient patient numbers, or other technical faults have led to some level of mistrust in the clinical trials on the use of Bacterial Lysates in a clinical setting.

High-quality, large, multicenter, double-blind, placebo-controlled, randomized clinical trials should be performed to establish the actual effects of immunostimulants and the effects of individual immunostimulant preparations, selecting patients by age, risk of RTI, comorbidities, and route of administration.

## Figures and Tables

**Table 1:** Characteristic commercial Bacterial Lysates used for treating respiratory tract infections.

Alkaline lysis		
Broncho-Vaxom (OM-85 BV)	*Haemophilus influenzae* *Klebsiella pneumoniae* *Klebsiella ozaenae* *Moraxella catarrhalis* *Staphylococcus aureus* *Streptococcus pneumoniae* *Streptococcus pyogenes* *Streptococcus viridans*	Oral capsules
Liuvac LW50020	*Haemophilus influenzae* *Klebsiella pneumoniae* *Moraxella catarrhalis* *Staphylococcus aureus* *Streptococcus pneumoniae* *Streptococcus pyogenes* *Streptococcus mitis*	Oral Tablets
Lantigen B	*Haemophilus influenzae* *Klebsiella pneumoniae* *Moraxella catarrhalis* *Staphylococcus aureus* *Streptococcus pneumoniae* *Streptococcus pyogenes*	Sublingual Drops
Mechanical lysis		
Ismigen PBML	*Haemophilus influenzae* *Klebsiella pneumoniae* *Klebsiella ozaenae* *Moraxella catarrhalis* *Staphylococcus aureus* *Streptococcus pneumoniae* *Streptococcus pyogenes* *Streptococcus viridans*	Sublingual tablets
Ribomunyl D53 Ribosomal–proteoglycan	*Haemophilus influenzae* *Klebsiella pneumoniae* *Streptococcus pneumoniae* *Streptococcus pyogenes*	Oral tablets or granules

## References

[1] World Health Organization (2017). The Top 10 Causes of Death.

[2] Jin X., Ren J., Li R., Gao Y., Zhang H., Li J., Zhang J., Wang X., Wang G. (2021). Global burden of upper respiratory infections in 204 countries and territories, from 1990 to 2019. EClinicalMedicine.

[3] GBD 2019 LRI Collaborators (2022). Age-sex differences in the global burden of lower respiratory infections and risk factors, 1990–2019: Results from the Global Burden of Disease Study 2019. Lancet Infect Dis..

[4] GBD 2016 Lower Respiratory Infections Collaborators (2018). Estimates of the global, regional, and national morbidity, mortality, and aetiologies of lower respiratory infections in 195 countries, 1990–2016: A systematic analysis for the Global Burden of Disease Study 2016. Lancet Infect Dis..

[5] Kollmann T.R., Kampmann B., Mazmanian S.K., Marchant A., Levy O. (2017). Protecting the newborn and young infant from infectious diseases: Lessons from immune ontogeny. Immunity.

[6] Zhang X., Zhivaki D., Lo-Man R. (2017). Unique aspects of the perinatal immune system. Nat. Rev. Immunol..

[7] Borghesi A., Marzollo A., Michev A., Fellay J. (2020). Susceptibility to infection in early life: A growing role for human genetics. Hum. Genet..

[8] Beudeker C.R., Vijlbrief D.C., van Montfrans J.M., Rooijakkers S.H.M., van der Flier M. (2022). Neonatal sepsis and transient immunodeficiency: Potential for novel immunoglobulin therapies?. Front. Immunol..

[9] Aronen M., Viikari L., Kohonen I., Vuorinen T., Hämeenaho M., Wuorela M., Sadeghi M., Söderlund-Venermo M., Viitanen M., Jartti T. (2019). Respiratory tract virus infections in the elderly with pneumonia. BMC Geriatr..

[10] Meyer K.C. (2010). The role of immunity and inflammation in lung senescence and susceptibility to infection in the elderly. Semin. Respir. Crit. Care Med..

[11] Millett E.R., Quint J.K., Smeeth L., Daniel R.M., Thomas S.L. (2013). Incidence of community-acquired lower respiratory tract infections and pneumonia among older adults in the United Kingdom: A population-based study. PLoS ONE.

[12] Janssens J.P., Krause K.H. (2004). Pneumonia in the very old. Lancet Infect. Dis..

[13] Yu H., Feng Z., Uyeki T.M., Liao Q., Zhou L., Feng L., Ye M., Xiang N., Huai Y., Yuan Y. (2011). Risk factors for severe illness with 2009 pandemic influenza A (H1N1) virus infection in China. Clin. Infect Dis..

[14] Akhtar A., Hassali M.A.A., Zainal H., Ali I., Iqbal M.S., Khan A.H. (2021). Respiratory-tract infections among geriatrics: Prevalence and factors associated with the treatment outcomes. Ther. Adv. Respir. Dis..

[15] Van Asten L., Van den Wijngaard C., Van Pelt W., van de Kassteele J., Meijer A., van der Hoek W., Kretzschmar M., Koopmans M. (2012). Mortality attributable to 9 common infections: Significant effect of influenza A, respiratory syncytial virus, influenza B, norovirus, and parainfluenza in elderly persons. J. Infect. Dis..

[16] Kisiel M.A., Zhou X., Björnsson E., Holm M., Dahlman-Höglund A., Wang J., Svanes C., Norbäck D., Franklin K.A., Malinovschi A. (2021). The risk of respiratory tract infections and antibiotic use in a general population and among people with asthma. ERJ Open Res..

[17] Denholm R., van der Werf E.T., Hay A.D. (2020). Use of antibiotics and asthma medication for acute lower respiratory tract infections in people with and without asthma: Retrospective cohort study. Respir. Res..

[18] Sethi S., Murphy T.F. (2008). Infection in the pathogenesis and course of chronic obstructive pulmonary disease. N. Engl. J. Med..

[19] Matkovic Z., Miravitlles M. (2013). Chronic bronchial infection in COPD. Is there an infective phenotype?. Respir. Med..

[20] Miravitlles M., Marin A., Monsó E., Vilà S., de la Roza C., Hervás R., Esquinas C., García M., Millares L., Morera J. (2010). Color of sputum is a marker of bacterial colonization in COPD. Respir. Res..

[21] Murin S., Bilello K.S. (2005). Respiratory tract infections: Another reason not to smoke. Cleve Clin. J. Med..

[22] Flatby H.M., Rasheed H., Ravi A., Thomas L.F., Liyanarachi K.V., Afset J.E., DeWan A.T., Brumpton B.M., Hveem K., Åsvold B.O. (2022). Risk of lower respiratory tract infections: A genome-wide association study with Mendelian randomization analysis in three independent European populations. Clin. Microbiol. Infect..

[23] Sabetta J.R., DePetrillo P., Cipriani R.J., Smardin J., Burns L.A., Landry M.L. (2010). Serum 25-hydroxyvitamin D and the incidence of acute viral respiratory tract infections in healthy adults. PLoS ONE.

[24] Matthews C.E., Ockene I.S., Freedson P.S., Rosal M.C., Merriam P.A., Hebert J.R. (2002). Moderate to vigorous physical activity and risk of upper-respiratory tract infection. Med. Sci. Sports Exerc..

[25] Almirall J., Bolíbar I., Serra-Prat M., Roig J., Hospital I., Carandell E., Agustí M., Ayuso P., Estela A., Torres A. (2008). New evidence of risk factors for community-acquired pneumonia: A population-based study. Eur. Respir. J..

[26] Kim J.L., Henneberger P.K., Lohman S., Olin A.C., Dahlman-Höglund A., Andersson E., Torén K., Holm M. (2016). Impact of occupational exposures on exacerbation of asthma: A population-based asthma cohort study. BMC Pulm. Med..

[27] Jakiela B., Brockman-Schneider R., Amineva S., Lee W.M., Gern J.E. (2008). Basal cells of differentiated bronchial epithelium are more susceptible to rhinovirus infection. Am. J. Respir. Cell Mol. Biol..

[28] Kaspersen K.A., Pedersen O.B., Petersen M.S., Hjalgrim H., Rostgaard K., Møller B.K., Juul-Sørensen C., Kotzé S., Dinh K.M., Erikstrup L.T. (2015). Obesity and risk of infection: Results from the Danish Blood Donor Study. Epidemiology.

[29] Muller L.M., Gorter K.J., Hak E., Goudzwaard W.L., Schellevis F.G., Hoepelman A.I., Rutten G.E. (2005). Increased risk of common infections in patients with type 1 and type 2 diabetes mellitus. Clin. Infect. Dis..

[30] Aghamohammadi A., Cheraghi T., Gharagozlou M., Movahedi M., Rezaei N., Yeganeh M., Parvaneh N., Abolhassani H., Pourpak Z., Moin M. (2009). IgA deficiency: Correlation between clinical and immunological phenotypes. J. Clin. Immunol..

[31] Bertrand Y., Sánchez-Montalvo A., Hox V., Froidure A., Pilette C. (2023). IgA-producing B cells in lung homeostasis and disease. Front. Immunol..

[32] Calder A.D., Perucca G., Johnson S.M., Pandey A.R., Moshal K., Kusters M.A. (2023). Lung infections in immunocompromised children. Pediatr. Radiol.

[33] Lehman H.K., Yu K.O.A., Towe C.T., Risma K.A. (2022). Respiratory Infections in Patients with Primary Immunodeficiency. J. Allergy Clin. Immunol. Pract..

[34] Mayaud C., Parrot A., Cadranel J. (2002). Pyogenic bacterial lower respiratory tract infection in human immunodeficiency virus-infected patients. Eur. Respir. J..

[35] Ashley E.A., Johnson M.A., Lipman M.C. (2000). Human immunodeficiency virus and respiratory infection. Curr. Opin. Pulm. Med..

[36] van de Vosse E., van Ostaijen-Ten Dam M.M., Vermaire R., Verhard E.M., Waaijer J.L., Bakker J.A., Bernards S.T., Eibel H., van Tol M.J., van Dissel J.T. (2017). Recurrent respiratory tract infections (RRTI) in the elderly: A late onset mild immunodeficiency?. Clin. Immunol..

[37] Barton J., Barton C., Bertoli L. (2019). Duration of frequent or severe respiratory tract infection in adults before diagnosis of IgG subclass deficiency. PLoS ONE.

[38] Pecego A.C., Amâncio R.T., Costa D.M., Bozza F.A., Siqueira M.M., Oliveira M.L., Cerbino-Neto J., Japiassu A. (2020). Etiology, clinical, and epidemiological characteristics of severe respiratory infection in people living with HIV. Int. J. STD AIDS.

[39] Pérez A., Montoro J., Hernani R., Lorenzo I., Hernández-Boluda J.C., Giménez E., Gómez M.D., Balaguer-Roselló A., Gonzalez-Barberá E., Guerreiro M. (2020). Assessment of immunodeficiency scoring index performance in enterovirus/rhinovirus respiratory infection after allogeneic hematopoietic stem cell transplantation. Transpl. Infect. Dis..

[40] Ciofu O., Hansen C.R., Høiby N. (2013). Respiratory bacterial infections in cystic fibrosis. Curr. Opin. Pulm. Med..

[41] Jones K.L., Adam M.P. (2015). Evaluation and diagnosis of the dysmorphic infant. Clin. Perinatol..

[42] Norlander T., Brönnegård M., Stierna P. (1999). The relationship of nasal polyps, infection, and inflammation. Am. J. Rhinol..

[43] Lindbaek M. (2006). Prescribing antibiotics to patients with acute cough and otitis media. Br. J. Gen. Pract..

[44] Raghav H., Tayal P., Das R., Mehta D.K. (2022). Appropriate Use of Antibiotics for the Management of Respiratory Tract Infections. Infect. Disord. Drug Targets..

[45] Ashworth M., Charlton J., Ballard K., Latinovic R., Gulliford M. (2005). Variations in antibiotic prescribing and consultation rates for acute respiratory infection in UK general practices 1995–2000. Br. J. Gen. Pract..

[46] Ventola C.L. (2015). The antibiotic resistance crisis: Part 1: Causes and threats. Pharm. Ther..

[47] Ventola C.L. (2015). The antibiotic resistance crisis: Part 2: Management strategies and new agents. Pharm. Ther..

[48] Peng Z., He W.Q., Hayen A., Hall J., Liu B. (2023). After-hours consultations and antibiotic prescribing for self-limiting upper respiratory tract infections in primary-care practices. Infect. Control Hosp. Epidemiol..

[49] van Houten C.B., Cohen A., Engelhard D., Hays J.P., Karlsson R., Moore E., Fernández D., Kreisberg R., Collins L.V., de Waal W. (2019). Antibiotic misuse in respiratory tract infections in children and adults-a prospective, multicentre study (TAILORED Treatment). Eur. J. Clin. Microbiol. Infect. Dis..

[50] Assoni L., Girardello R., Converso T.R., Darrieux M. (2021). Current Stage in the Development of *Klebsiella pneumoniae* Vaccines. Infect. Dis. Ther..

[51] Cazzola M., Rogliani P., Curradi G. (2008). Bacterial extracts for the prevention of acute exacerbations in chronic obstructive pulmonary disease: A point of view. Respir. Med..

[52] Esposito S., Soto-Martinez M.E., Feleszko W., Jones M.H., Shen K.L., Schaad U.B. (2018). Nonspecific immunomodulators for recurrent respiratory tract infections, wheezing and asthma in children. Curr. Opin. Allergy Clin. Immunol..

[53] Cazzola M., Anapurapu S., Page C.P. (2012). Polyvalent mechanical bacterial lysate for the prevention of recurrent respiratory infections: A meta-analysis. Pulmon. Pharmacol. Ther..

[54] Hancock R.E.W., Nijnik A., Philpott D.J. (2012). Modulating immunity as a therapy for bacterial infections. Nat. Rev. Microbiol..

[55] https://www.ema.europa.eu/en/medicines/human/referrals/bacterial-lysates-containing-medicinal-products-indicated-respiratory-conditions.

[56] Suárez N., Ferrara F., Rial A., Dee V., Chabalgoity J.A. (2020). Bacterial Lysates as Immunotherapies for Respiratory Infections: Methods of Preparation. Front. Bioeng. Biotechnol..

[57] Guani-Guerra E., Negrete-Garcia M.C., Montes-Vizuet R., Asbun-Bojalil J., Teran L.M. (2011). Human beta-defensin-2 induction in nasal mucosa after administration of bacterial lysates. Arch. Med. Res..

[58] Bienenstock J., McDermott M., Befus D., O’Neill M. (1978). A common mucosal immunologic system involving the bronchus, breast and bowel. Adv. Exp. Med. Biol..

[59] Rossi G.A., Pohunek P., Feleszko W., Ballarini S., Colin A.A. (2020). Viral infections and wheezing-asthma inception in childhood: Is there a role for immunomodulation by oral bacterial lysates?. Clin. Transl. Allergy.

[60] Esposito S., Jones M.H., Feleszko W., Martell J.A.O., Falup-Pecurariu O., Geppe N., Martinón-Torres F., Shen K.L., Roth M., Principi N. (2020). Prevention of new respiratory episodes in children with recurrent respiratory infections: An expert consensus statement. Microorganisms.

[61] Manolova V., Flace A., Jeandet P., Bessler W.C., Pasquali C. (2017). Biomarkers induced by the immunomodulatory bacterial extract OM-85: Unique roles for Peyer’s Patches and intestinal epithelial cells. J. Clin. Cell Immunol..

[62] Parola C., Salogni L., Vaira X., Scutera S., Somma P., Salvi V., Musso T., Tabbia G., Bardessono M., Pasquali C. (2013). Selective activation of human dendritic cells by OM-85 through a NF-kB and MAPK dependent pathway. PLoS ONE.

[63] Huber M., Mossmann H., Bessler W.G. (2005). Th1-orientated immunological properties of the bacterial extract OM-85-BV. Eur. J. Med. Res..

[64] Ferrara F., Rial A., Suárez N., Chabalgoity J.A. (2021). Polyvalent Bacterial Lysate Protects Against Pneumonia Independently of Neutrophils, IL-17A or Caspase-1 Activation. Front. Immunol..

[65] Marit M., Chiavaroli C. (2007). Mechanism of action and therapeutic efficacy of the biotechnology-derived immunostimulating extract OM85 in respiratory tract infections. Int. J. Biotechnol..

[66] Bartkowiak-Emeryk M., Emeryk A., Roliński J., Wawryk-Gawda E., Markut-Miotła E. (2021). Impact of Polyvalent Mechanical Bacterial Lysate on lymphocyte number and activity in asthmatic children: A randomized controlled trial. Allergy Asthma Clin. Immunol..

[67] Lee Y.K., Haam J.H., Suh E., Cho S.H., Kim Y.S. (2022). A Case-Control Study on the Changes in Natural Killer Cell Activity following Administration of Polyvalent Mechanical Bacterial Lysate in Korean Adults with Recurrent Respiratory Tract Infection. J. Clin. Med..

[68] Bosco A. (2023). Emerging role for interferons in respiratory viral infections and childhood asthma. Front. Immunol..

[69] Dang A.T., Pasquali C., Ludigs K., Guarda G. (2017). OM-85 is an immunomodulator of interferon-β production and inflammasome activity. Sci. Rep..

[70] Rial A., Ferrara F., Suárez N., Scavone P., Marqués J.M., Chabalgoity J.A. (2016). Intranasal administration of a polyvalent bacterial lysate induces self-restricted inflammation in the lungs and a Th1/Th17 memory signature. Microbes Infect..

[71] Sidoti Migliore G., Pezzino G., Cavaliere R., De Pasquale C., Ferlazzo G. (2021). On immunostimulants and dendritic cell activation. Immunol. Lett..

[72] Macri C., Fancke B., Radford K.J., O’Keeffe M. (2019). Monitoring Dendritic Cell Activation and Maturation. Methods Mol. Biol..

[73] Zelle-Rieser C., Ramoner R., Bartsch G., Thurnher M. (2001). A Clinically Approved Oral Vaccine Against Pneumotropic Bacteria Induces the Terminal Maturation of Cd83+ Immunostimulatory Dendritic Cells. Immunol. Lett..

[74] Pasquali C., Salami O., Taneja M., Gollwitzer E.S., Trompette A., Pattaroni C., Yadava K., Bauer J., Marsland B.J. (2014). Enhanced Mucosal Antibody Production and Protection Against Respiratory Infections Following an Orally Administered Bacterial Extract. Front. Med..

[75] Spisek R., Brazova J., Rozkova D., Zapletalova K., Sediva A., Bartunkova J. (2004). Maturation of Dendritic Cells by Bacterial Immunomodulators. Vaccine.

[76] Gaudino S.J., Kumar P. (2019). Cross-Talk Between Antigen Presenting Cells and T Cells Impacts Intestinal Homeostasis, Bacterial Infections, and Tumorigenesis. Front. Immunol..

[77] Koatz A.M., Coe N.A., Ciceran A., Alter A.J. (2016). Clinical and immunological benefits of OM-85 bacterial lysate in patients with allergic rhinitis, asthma, and COPD and recurrent respiratory infections. Lung.

[78] Rossi G.A., Peri C., Raynal M.E., Defilippi A.C., Risso F.M., Schenone G., Pallestrini E., Melioli G. (2003). Naturally occurring immune response against bacteria commonly involved in upper respiratory tract infections: Analysis of the antigen-specific salivary IgA levels. Immunol. Lett..

[79] Ruedl C., Fruhwirth M., Wick G., Wolf H. (1994). Immune response in the lungs following oral immunization with bacterial lysates of respiratory pathogens. Clin. Diagn. Lab. Immunol..

[80] Kearney S.C., Dziekiewicz M., Feleszko W. (2015). Immunoregulatory and immunostimulatory responses of bacterial lysates in respiratory infections and asthma. Ann. Allergy Asthma Immunol..

[81] Cleaver J.O., You D., Michaud D.R., Pruneda F.A., Juarez M.M., Zhang J., Weill P.M., Adachi R., Gong L., Moghaddam S.J. (2014). Lung epithelial cells are essential effectors of inducible resistance to pneumonia. Mucosal Immunol..

[82] Sidoti Migliore G., Campana S., Barberi C., De Pasquale C., Pezzino G., Cavaliere R., Orecchia P., Ginestra G., Mandalari G., Del Zotto G. (2023). Mechanical bacterial lysate enhances antimicrobial barrier mechanisms in human airway epithelial cells. J. Leukoc. Biol..

[83] Ballarini S., Ardusso L., Ortega Martell J.A., Sacco O., Feleszko W., Rossi G.A. (2022). Can bacterial lysates be useful in prevention of viral respiratory infections in childhood? The results of experimental OM-85 studies. Front. Pediatr..

[84] Liu Y.W., Dong S.H., Zhan G.Y., Tan H.Z., Peng Y.Q., Wei F. (2017). Analysis of the effect of bacterial lysate and the immunologic mechanism in treating infant bronchiolitis. Eur. Rev. Med. Pharmacol. Sci..

[85] Lanzilli G., Traggiai E., Braido F., Garelli V., Folli C., Chiappori A., Riccio A.M., Bazurro G., Agazzi A., Magnani A. (2013). Administration of a polyvalent mechanical bacterial lysate to elderly patients with COPD: Effects on circulating T, B and NK cells. Immunol. Lett..

[86] Bosch A.A., Biesbroek G., Trzcinski K., Sanders E.A., Bogaert D. (2013). Viral and bacterial interactions in the upper respiratory tract. PLoS Pathog..

[87] Sánchez-Ramón S., Conejero L., Netea M.G., Sancho D., Palomares Ó., Subiza J.L. (2018). Trained Immunity-Based Vaccines: A New Paradigm for the Development of Broad-Spectrum Anti-infectious Formulations. Front. Immunol..

[88] Bindu S., Dandapat S., Manikandan R., Dinesh M., Subbaiyan A., Mani P., Dhawan M., Tiwari R., Bilal M., Emran T.B. (2022). Prophylactic and therapeutic insights into trained immunity: A renewed concept of innate immune memory. Hum. Vaccin. Immunother..

[89] Randall T.D. (2010). Bronchus-associated lymphoid tissue (BALT) structure and function. Adv. Immunol..

[90] Halle S., Dujardin H., Bakocevic N., Fleige H., Danzer H., Willenzon S., Suezer Y., Hämmerling G., Garbi N. (2009). Induced bronchus-associated lymphoid tissue serves as a general priming site for T cells and is maintained by dendritic cells. J. Exp. Med..

[91] Silva-Sanchez A., Randall T.D. (2020). Role of iBALT in Respiratory Immunity. Curr. Top. Microbiol. Immunol..

[92] Mestecky J. (1987). The common mucosal immune system and current strategies for induction of immune responses in external secretions. J. Clin. Immunol..

[93] Olszewski W.L. (1986). Lymphatics, lymph and lymphoid cells: An integrated immune system. Eur. Surg. Res..

[94] Bessler W.G., Vor dem Esche U., Masihi N. (2010). The bacterial extract OM-85 BV protects mice against influenza and Salmonella infection. Int. Immunopharmacol..

[95] Rossi G.A., Bessler W., Ballarini S., Pasquali C. (2018). Evidence that a primary anti-viral stimulation of the immune response by OM-85 reduces susceptibility to a secondary respiratory bacterial infection in mice. Ital. J. Pediatr..

[96] Roth M., Pasquali C., Stolz D., Tamm M. (2017). Broncho Vaxom (OM-85) modulates rhinovirus docking proteins on human airway epithelial cells via Erk1/2 mitogen activated protein kinase and cAMP. PLoS ONE.

[97] Coviello S., Wimmenauer V., Polack F.P., Irusta P.M. (2014). Bacterial lysates improve the protective antibody response against respiratory viruses through Toll-like receptor 4. Hum. Vaccin. Immunother..

[98] Khameneh H.J., Bolis M., Ventura P.M.O., Cassanmagnago G.A., Fischer B.A., Zenobi A., Guerra J., Buzzago I., Bernasconi M., Zaman G.J.R. (2024). The bacterial lysate OM-85 engages Toll-like receptors 2 and 4 triggering an immunomodulatory gene signature in human myeloid cells. Mucosal Immunol..

[99] Antunes K.H., Cassão G., Santos L.D., Borges S.G., Poppe J., Gonçalves J.B., Nunes E.D.S., Recacho G.F., Sousa V.B., Da Silva G.S. (2022). Airway Administration of Bacterial Lysate OM-85 Protects Mice Against Respiratory Syncytial Virus Infection. Front. Immunol..

[100] Pivniouk V., Pivniouk O., DeVries A., Uhrlaub J.L., Michael A., Pivniouk D., VanLinden S.R., Conway M.Y., Hahn S., Malone S.P. (2022). The OM-85 bacterial lysate inhibits SARS-CoV-2 infection of epithelial cells by downregulating SARS-CoV-2 receptor expression. J. Allergy Clin. Immunol..

[101] Navarro S., Cossalter G., Chiavaroli C., Kanda A., Fleury S., Lazzari A., Lazzari A., Cazareth J., Sparwasser T., Dombrowicz D. (2011). The Oral Administration of Bacterial Extracts Prevents Asthma Via the Recruitment of Regulatory T Cells to the Airways. Mucosal Immunol..

[102] Fu R., Li J., Zhong H., Yu D., Zeng X., Deng M., Sun Y., Wen W., Li H. (2014). Broncho-Vaxom attenuates allergic airway inflammation by restoring GSK3β-related T regulatory cell insufficiency. PLoS ONE.

[103] Zhong H., Wei J., Yao Y., Fu R., Li H., Fu Q., Wen W. (2017). A bacterial extract of OM-85 Broncho-Vaxom suppresses ovalbumin-induced airway inflammation and remodeling in a mouse chronic allergic asthma model. Int. J. Clin. Exp. Pathol..

[104] Pivniouk V., Gimenes-Junior J.A., Ezeh P., Michael A., Pivniouk O., Hahn S., VanLinden S.R., Malone S.P., Abidov A., Anderson D. (2022). Airway administration of OM-85, a bacterial lysate, blocks experimental asthma by targeting dendritic cells and the epithelium/IL-33/ILC2 axis. J. Allergy Clin. Immunol..

[105] Liu C., Huang R., Yao R., Yang A. (2017). The Immunotherapeutic Role of Bacterial Lysates in a Mouse Model of Asthma. Lung.

[106] Zagar S., Lofler-Badzek D. (1988). Broncho-Vaxom in children with rhinosinusitis: A double-blind clinical trial. ORL J. Otorhinolaryngol. Relat. Spec..

[107] Collet J.P., Ducruet T., Kramer M.S., Haggerty J., Floret D., Chomel J.J., Durr F. (1993). Stimulation of nonspecific immunity to reduce the risk of recurrent infections in children attending day-care centers. The Epicreche Research Group. Pediatr. Infect. Dis. J..

[108] Gomez B.D., De la T.C., Alvarez A., Faure A., Berber A. (1998). Safety and efficacy of OM-85-BV plus amoxicillin/clavulanate in the treatment of sub- acute sinusitis and the prevention of recurrent infections in children. Allergol. Immunopathol..

[109] Sramek J., Josifko M., Helcl J., Holoubková E., Janout V., Kozesník B., Macátová I. (1986). Bacterial lysate (I.R.S. 19) applied intranasally in the prevention of acute respiratory diseases in children: A randomized double-blind study. J. Hyg. Epidemiol. Microbiol. Immunol..

[110] Ruah S.B., Ruah C., van Aubel A., Abel S., Elsasser U. (2001). Efficacy of a polyvalent bacterial lysate in children with recurrent respiratory tract infections. Adv. Ther..

[111] Gutierrez-Tarango M.D., Berber A. (2001). Safety and efficacy of two courses of OM-85 BV in the prevention of respiratory tract infections in children during 12 months. Chest.

[112] Jara-Perez J.V., Berber A. (2000). Primary prevention of acute respiratory tract infections in children using a bacterial immunostimulant: A double-masked, placebo-controlled clinical trial. Clin. Ther..

[113] Mora R., Salzano F.A., Mora E., Guastini L. (2012). Efficacy of a topical suspension of bacterial antigens for the management of chronic suppurative otitis media. Eur. Arch. Otorhinolaryngol..

[114] Su X.O., Li Y.H., Qian S.Y., Liang Y.X. (2013). Clinical curative effect of Lantigen B on bronchial asthma children with recurrent respiratory tract infection. Mod. Prev. Med. (Artic. Chin.).

[115] Chen A.H., Chen R.C., Zhang C.Q. (2004). Efficacy of sublingual polyvalent bacterial vaccine (Lantigen B) in children with recurrent respiratory infection: A randomized double-blind controlled clinical trial. Zhonghua Er Ke Za Zhi [Chin. J. Pediatr.].

[116] Schaad U.B., Mutterlein R., Goffin H., BV-Child Study Group (2002). Immunostimulation with OM-85 in children with recurrent infections of the upper respiratory tract: A double-blind, placebo-controlled multicenter study. Chest.

[117] Del-Rio-Navarro B.E., Luis Sienra-Monge J.J., Berber A., Torres-Alcántara S., Avila-Castañón L., Gómez-Barreto D. (2003). Use of OM-85 BV in children suffering from recurrent respiratory tract infections and subnormal IgG subclass levels. Allergol. Immunopathol..

[118] Rosaschino F., Cattaneo L. (2004). Strategies for optimizing compliance of paediatric patients for seasonal antibacterial vaccination with sublingually administered polyvalent mechanical bacterial lysates (PMBL). Acta Biomed. Ateneo Parm..

[119] Bitar M.A., Saade R. (2013). The role of OM-85 BV (Broncho-Vaxom) in preventing recurrent acute tonsillitis in children. Int. J. Pediatr. Otorhinolaryngol..

[120] Esposito S., Bianchini S., Bosis S., Tagliabue C., Coro I., Argentiero A., Principi N. (2019). A randomized, placebo-controlled, double-blinded, single-centre, phase IV trial to assess the efficacy and safety of OM-85 in children suffering from recurrent respiratory tract infections. J. Transl. Med..

[121] Rebolledo L., Rodríguez-Vigil C., Carmen L., Llorente E., Guallar M., Villoria J., Vicente E. (2023). Bacterial immunotherapy is highly effective in reducing recurrent upper respiratory tract infections in children: A prospective observational study. Eur. Arch. Otorhinolaryngol..

[122] Byl B., Libin M., Gerard M., Clumeck N., Goldman M., Mascart-Lemone F. (1998). Bacterial Extract Om85-Bv Induces Interleukin-12-Dependent IFN-Gamma Production by Human Cd4+ T Cells. J. Interferon Cytokine Res..

[123] Lanzilli G., Falchetti R., Tricarico M., Ungheri D., Fuggetta M.P. (2005). In Vitro Effects of an Immunostimulating Bacterial Lysate on Human Lymphocyte Function. Int. J. Immunopathol. Pharmacol..

[124] Mijanur Rahman M., Darren Grice I., Ulett G.C., Wei M.Q. (2024). Advances in Bacterial Lysate Immunotherapy for Infectious Diseases and Cancer. J Immunol. Res..

[125] Peebles R.S., Aronica M.A. (2019). Proinflammatory Pathways in the Pathogenesis of Asthma. Clin. Chest Med..

[126] Braga M., Quecchia C., Cavallucci E., Di Giampaolo L., Schiavone C., Petrarca C., Di Gioacchino M. (2011). T regulatory cells in allergy. Int. J. Immunopathol. Pharmacol..

[127] Thomas A.O., Lemanske R.F., Jackson D.J. (2014). Infections and their role in childhood asthma inception. Pediatr. Allergy Immunol..

[128] Lan F., Zhang N., Gevaert E., Zhang L., Bachert C. (2016). Viruses and bacteria in Th2-biased allergic airway disease. Allergy.

[129] Razi C.H., Harmanci K., Abaci A., Özdemir O., Hızlı S., Renda R., Keskin F. (2010). The immunostimulant OM-85 BV prevents wheezing attacks in preschool children. J. Allergy Clin. Immunol..

[130] Paupe J., Paupe G. (1986). Biostim prevention of recurrent respiratory infections in children. A double-blind versus placebo study. Ann. Pediatr..

[131] Lu Y., Li Y., Xu L., Xia M., Cao L. (2015). Bacterial lysate increases the percentage of natural killer T cells in peripheral blood and alleviates asthma in children. Pharmacology.

[132] Feleszko W., Marengo R., Vieira A.S., Ratajczak K., Mayorga Butrón J.L. (2019). Immunity-targeted approaches to the management of chronic and recurrent upper respiratory tract disorders in children. Clin. Otolaryngol..

[133] Sly P.D., Galbraith S., Islam Z., Holt B., Troy N., Holt P.G. (2019). Primary prevention of severe lower respiratory illnesses in at-risk infants using the immunomodulator OM-85. J. Allergy Clin. Immunol..

[134] Del-Rio-Navarro B.E., Espinosa R.F., Flenady V., Sienra-Monge J.J. (2012). Immunostimulants for preventing respiratory tract infection in children (Review). Evid-Based Child. Health.

[135] Yin J., Xu B., Zeng X., Shen K. (2018). Broncho-Vaxom in pediatric recurrent respiratory tract infections: A systematic review and meta-analysis. Int. Immunopharmacol..

[136] Castro-Rodriguez J.A., Turi K.N., Forno E. (2024). A critical analysis of the effect of OM-85 for the prevention of recurrent respiratory tract infections or wheezing/asthma from systematic reviews with meta-analysis. Pediatr. Allergy Immunol..

[137] Braido F., Melioli G., Nicolini G., Ferraris M., Di Girolamo S., Di Gioacchino M., Canonica G.W. (2024). Sublingually administered bacterial lysates: Rationale, mechanisms of action and clinical outcomes. Drugs Context..

[138] Berber A., Del-Río-Navarro B.E., Reyes-Noriega N., Sienra-Monge J.J.L. (2022). Immunostimulants for preventing respiratory tract infection in children: A systematic review and meta-analysis. World Allergy Organ. J..

[139] Bouquet J., Tabor D.E., Silver J.S., Nair V., Tovchigrechko A., Griffin M.P., Esser M.T., Sellman B.R., Jin H. (2020). Microbial burden and viral exacerbations in a longitudinal multicenter COPD cohort. Respir. Res..

[140] Mulpuru S., Andrew M.K., Ye L., Hatchette T., LeBlanc J., El-Sherif M., MacKinnon-Cameron D., Aaron S.D., Alvarez G.G., Forster A.J. (2022). Serious Outcomes Surveillance and Canadian Immunization Research Network (CIRN) Investigators. Impact of respiratory viral infections on mortality and critical illness among hospitalized patients with chronic obstructive pulmonary disease. Influenza Other Respir. Viruses.

[141] Love M.E., Proud D. (2022). Respiratory Viral and Bacterial Exacerbations of COPD-The Role of the Airway Epithelium. Cells.

[142] Debbas N., Derenne J.P. (1990). Preventive effects of an immunostimulating product on recurrent infections of chronic bronchitis in the elderly. Lung.

[143] Xinogalos S., Duratsos D., Varonos D. (1993). Clinical effectiveness of Broncho-vaxom (BV) in patients with chronic obstructive pulmonary disease. J. Immunother..

[144] Collet J.P., Shapiro P., Ernst P., Renzi T., Ducruet T., Robinson A. (1997). Effects of an immunostimulating agent on acute exacerbations and hospitalizations in patients with chronic obstructive pulmonary disease. The PARI-IS Study Steering Committee and Research Group. Prevention of Acute Respiratory Infection by an Immunostimulant. Am. J. Respir. Crit. Care Med..

[145] Li J., Zheng J.P., Yuan J.P., Zeng G.Q., Zhong N.S., Lin C.Y. (2004). Protective effect of a bacterial extract against acute exacerbation in patients with chronic bronchitis accompanied by chronic obstructive pulmonary disease. Chin. Med. J..

[146] Solèr M., Mütterlein R., Cozma G. (2007). Double-blind study of OM-85 in patients with chronic bronchitis or mild chronic obstructive pulmonary disease. Respiration.

[147] Cazzola M., Noschese P., Di Perna F. (2009). Value of adding a polyvalent mechanical bacterial lysate to therapy of COPD patients under regular treatment with salmeterol/fluticasone. Ther. Adv. Respir. Dis..

[148] Genda A.M., Davidescu L., Ulmeanu R., Ilisie M. (2014). Value of adding treatment with lyophilized bacterial lysates in reducing COPD exacerbations in COPD patients risk group C and D. Eur. Respir. J..

[149] Braido F., Melioli G., Cazzola M., Fabbri L., Blasi F., Moretta L., Canonica G.W., AIACE Study Group (2015). Sub-lingual administration of a polyvalent mechanical bacterial lysate (PMBL) in patients with moderate, severe, or very severe chronic obstructive pulmonary disease (COPD) according to the GOLD spirometric classification: A multicentre, double-blind, randomised, controlled, phase IV study (AIACE study: Advanced immunological approach in copd exacerbation). Pulm. Pharmacol. Ther..

[150] Pan L., Jiang X.G., Guo J., Tian Y., Liu C.T. (2015). Effects of OM-85 BV in patients with chronic obstructive pulmonary disease: A systematic review and meta-analysis. J. Clin. Pharmacol..

[151] Tang H., Fang Z., Saborío G.P., Xiu Q. (2015). Efficacy and safety of OM-85 in patients with chronic bronchitis and/or chronic obstructive pulmonary disease. Lung.

[152] Zeng D., Huang J., Wang B., Chen G. (2019). Efficacy of Broncho-Vaxom on chronic obstructive pulmonary disease in elderly patients. Chin. J. Geriatr..

[153] Avdeev S.N., Nuralieva G.S., Gainitdinova V.V., Baimakanova G.E., So A.K., Merzhoeva Z.M. (2020). Clinical efficacy of mechanical bacterial lysate in the prevention of infectious exacerbations of chronic obstructive pulmonary disease. Ter. Arkh..

[154] Ricci R., Palmero C., Bazurro G., Riccio A.M., Garelli V., Di Marco E., Cirillo C., Braido F., Canonica G.W., Melioli G. (2014). The administration of a polyvalent mechanical bacterial lysate in elderly patients with COPD results in serological signs of an efficient immune response associated with a reduced number of acute episodes. Pulm. Pharmacol. Ther..

[155] Huang Y., Pei Y., Qian Y., Yao Z., Chen C., Du J., Shi M., Zhou T. (2022). A Meta-Analysis on the Efficacy and Safety of Bacterial Lysates in Chronic Obstructive Pulmonary Disease. Front. Med..

[156] Ahrens J., Wiedenbach M. (1984). Efficacy of the immunostimulant Broncho-Vaxom. Schweiz. Med. Wochenschr..

[157] Debelic M., Eckenberger H.P. (1992). Prevention of recurrent infection of the upper and lower airways. Multicenter, open study over three months. Fortschr. Med..

[158] Rutishauser M., Pitzke P., Grevers G., van Aubel A., Elsasser U., Kämmereit A. (1998). Use of a polyvalent bacterial lysate in patients with recurrent respiratory tract infections: Results of a prospective, placebo-controlled, randomized, double-blind study. Adv. Ther..

[159] Alecsandru D., Valor L., Sanchez-Ramon S., Gil J., Carbone J., Navarro J., Rodríguez J., Rodríguez-Sainz C., Fernández-Cruz E. (2011). Sublingual Therapeutic Immunization With a Polyvalent Bacterial Preparation in Patients With Recurrent Respiratory Infections: Immunomodulatory Effect on Antigen-Specific Memory Cd4+ T Cells and Impact on Clinical Outcome. Clin. Exp. Immunol..

[160] Heintz B., Schlenter W.W., Kirsten R., Nelson K. (1989). Clinical efficacy of Broncho-Vaxom in adult patients with chronic purulent sinusitis—A multi-centric, placebo-controlled, double-blind study. Int. J. Clin. Pharmacol. Ther. Toxicol..

[161] Tielemans C., Gastaldello K., Husson C., Marchant A., Delville J.P., Vanherweghem J.L., Goldman M. (1999). Efficacy of oral immunotherapy on respiratory infections in hemodialysis patients: A double-blind, placebo-controlled study. Clin. Nephrol..

[162] Macchi A., Vecchia L.D. (2005). Open comparative, randomized control- led clinical study of a new immunostimulating bacterial lysate in the prophylaxis of upper respiratory tract infections. Arzneimittelforschung.

[163] Zhang M., Luan H., Zhang Q., Wang L., Lv Y.M., He F., Chen Y., Zeng H.B., Yao Y., Liu Q. (2012). Prevention of infection in immunosuppressive patients with autoimmune nephrosis by using an immunostimulating bacterial lysate Broncho-Vaxom. Hum. Vaccin. Immunother..

[164] Capetti A., Cossu M.V., Carenzi L., Rizzardini G. (2013). Four years of immunization with OM-85 BV to prevent respiratory infections in HIV+ patients. Hum. Vaccin. Immunother..

[165] Tricarico D., Varricchio A., D’Ambrosio S., Ascione E., Motta G. (2004). Prevention of recurrent upper respiratory tract infections in a community of cloistered nuns using a new immunostimulating bacterial lysate. A randomized, double-blind clinical trial. Arzneimittelforschung.

[166] Braido F., Melioli G., Candoli P., Cavalot A., Di Gioacchino M., Ferrero V., Incorvaia C., Mereu C., Ridolo E., Rolla G. (2014). The bacterial lysate Lantigen B reduces the number of acute episodes in patients with recurrent infections of the respiratory tract: The results of a double blind, placebo controlled, multicenter clinical trial. Immunol. Lett..

[167] Kostinov M.P., Gainitdinova V.V., Kazharova S.V., Vlasenko A.E., Polishchuk V.B., Mashilov K.V. (2023). Use of immunomodulatory therapy as part of comprehensive treatment of non-severe community-acquired pneumonia and its long-term results. Drugs Context..

[168] Pizzimenti P., D’Agostino A., Pirrello P., Ruiba A., Melioli G. (2023). The SARS-CoV-2 cellular receptor ACE2 is expressed in oropharyngeal cells and is modulated in vitro by the bacterial lysate Lantigen B. Arch. Clin. Biomed. Res..

[169] Braido F., Melioli G., Nicolini G., Canonica G.W. (2023). Prevention of recurrent respiratory tract infections: A literature review of the activity of the bacterial lysate Lantigen B. Eur. Rev. Med. Pharmacol. Sci..

[170] Chiapinotto S., Sarria E.E., Mocelin H.T., Lima J.A.B., Mattiello R., Fischer G.B. (2021). Impact of non-pharmacological initiatives for COVID-19 on hospital admissions due to pediatric acute respiratory illnesses. Paediatr. Respir. Rev..

[171] Uppala R., Sitthikarnkha P., Niamsanit S., Sutra S., Thepsuthammarat K., Techasatian L., Anantasit N., Teeratakulpisarn J. (2022). Effect of the COVID-19 Pandemic on Lower Respiratory Tract Infection Determinants in Thai Hospitalized Children: National Data Analysis 2015–2020. Trop. Med. Infect Dis..

[172] Macías Reyes M.J., Vidal-Alaball J., Suwezda E.A., Miró Catalina Q., Homs M., Ruiz-Comellas A. (2023). Prevalence of Respiratory Infections during the 2018–2020 Period in the Paediatric Population of Primary Care Centres in Central Catalonia. Healthcare.

[173] Center for Disease Control and Prevention, USA. https://www.cdc.gov/vaccines/vpd/pneumo/hcp/about-vaccine.html#:~:text=46%25%20effica-cy%20against%20vaccine%2Dtype,type%20non%2Dbacteremic%20pneumococcal%20pneumonia.

[174] Obolski U., Lourenço J., Thompson C., Thompson R., Gori A., Gupta S. (2018). Vaccination can drive an increase in frequencies of antibiotic resistance among nonvaccine serotypes of Streptococcus pneumoniae. Proc. Natl. Acad. Sci. USA.

[175] Smit J.J., Lindell D.M., Boon L., Kool L., Lambrecht B.M., Lukacs N.W. (2008). The balance between plasmacytoid DC versus conventional DC determines pulmonary immunity to virus infections. PLoS ONE.

[176] Kodama S., Suzuki M. (2011). Nasal-associated lymphoid tissue immunity and vaccine development. Adv. Otorhinolaryngol..

[177] Liao D., Su X., Wang J., Yu J., Luo H., Tian W., Ye Z., He J. (2023). Pushing the envelope: Immune mechanism and application landscape of macrophage-activating lipopeptide-2. Front. Immunol..

